# Tackling misinformation in mobile social networks a BERT-LSTM approach for enhancing digital literacy

**DOI:** 10.1038/s41598-025-85308-4

**Published:** 2025-01-07

**Authors:** Jun Wang, Xiulai Wang, Airong Yu

**Affiliations:** 1 School of Management Science and Engieering, Nanjing University of Information Science and Technology, Nanjing, China; 2 School of Artificial Intelligence, Nanjing Vocational College of Information Technology, Nanjing, China; 3 The Army Engineering University of PLA, Nanjing, 211117 Jiangsu China

**Keywords:** Psychology and behaviour, Sustainability

## Abstract

The rapid proliferation of mobile social networks has significantly accelerated the dissemination of misinformation, posing serious risks to social stability, public health, and democratic processes. Early detection of misinformation is essential yet challenging, particularly in contexts where initial content propagation lacks user feedback and engagement data. This study presents a novel hybrid model that combines Bidirectional Encoder Representations from Transformers (BERT) with Long Short-Term Memory (LSTM) networks to enhance the detection of misinformation using only textual content. Extensive evaluations revealed that the BERT-LSTM model achieved an accuracy of 93.51%, a recall of 91.96%, and an F1 score of 92.73% in identifying misinformation. A controlled user study with 100 participants demonstrated the model’s effectiveness as an educational tool, with the experimental group achieving 89.4% accuracy in misinformation detection compared to 74.2% in the control group, while showing increased confidence levels and reduced decision-making time. Beyond its technical efficacy, the model exhibits significant potential in fostering critical thinking skills necessary for digital literacy. The findings underscore the transformative potential of advanced AI techniques in addressing the challenges of misinformation in the digital age.

## Introduction

The proliferation of mobile social networks has dramatically accelerated the spread of misinformation, posing significant challenges to social stability, public health, and democratic processes^1,2^. The ease of access, broad user base, and rapid sharing capabilities inherent to these platforms create an environment where false information can quickly gain traction^3^. This phenomenon has become particularly concerning in recent years, with events such as the COVID-19 pandemic highlighting the potential real-world consequences of unchecked misinformation^4^.

Early detection of misinformation is crucial but challenging, particularly in mobile social networks where initial propagation often lacks user feedback and sharing data^5^. Traditional approaches to misinformation detection often rely on features that are not immediately available in the early stages of content dissemination, such as user engagement metrics or propagation patterns^6^. This limitation underscores the need for innovative methods that can effectively identify potential misinformation based solely on textual content.

Recent advancements in natural language processing (NLP) and deep learning have opened new avenues for addressing this challenge^7^. Particularly promising are transformer-based models like Bidirectional Encoder Representations from Transformers (BERT), which have demonstrated remarkable capabilities in understanding contextual nuances in text^8^. When combined with sequence processing architectures like Long Short-Term Memory (LSTM) networks, these models offer potential for more nuanced and accurate analysis of textual content^9^.

While significant progress has been made in developing sophisticated misinformation detection models, there remains a gap in translating these technical advancements into practical tools for enhancing digital literacy^10^. As the volume and complexity of online information continue to grow, empowering users with the skills to critically evaluate content becomes increasingly important^11–14^.

Given this context, our study aims to address three primary objectives:

1. To develop a robust early-stage detection model for misinformation in mobile social networks using only textual content.
2. To evaluate the performance of this model against conventional classifiers using standard metrics.
3. To explore the potential of the developed model as an educational tool for enhancing digital literacy.

By focusing on these objectives, we aim to advance the technical capabilities of misinformation detection while also contributing to the broader goal of fostering critical thinking skills within digital environments. This study leverages recent advancements in natural language processing (NLP) and deep learning to tackle the pressing issue of misinformation in mobile social networks^15^. By developing a model capable of effectively identifying potential misinformation based solely on textual content, we aim to provide a robust tool for early intervention. Furthermore, by exploring the educational potential of this model, we endeavor to contribute to the overarching goal of enhancing digital literacy in an era characterized by information overload.

The paper is structured as follows: “Literature review” section offers a comprehensive review of the existing literature on misinformation detection and digital literacy, emphasizing both advancements and ongoing challenges in these fields.  “Models and methods” section details our methodology, including a thorough description of the dataset, the architecture of the BERT-LSTM model, and the experimental setup used to evaluate its performance. “Conclusion” section presents the experimental results, providing a comparative analysis of the BERT-LSTM model’s performance relative to traditional classifiers.  “Effectiveness analysis” section discusses the broader implications of our findings, focusing on the technical efficacy of the model and its potential applications in enhancing digital literacy. “Discussion” section concludes the paper by summarizing the key findings and proposing directions for future research.

## Literature review

The proliferation of misinformation in mobile social networks presents a significant challenge to digital societies, necessitating a multifaceted approach that combines advanced computational techniques with strategies to enhance digital literacy. This review synthesizes current research across three key domains: misinformation detection in social networks, deep learning applications in natural language processing, and digital literacy interventions.

### Misinformation detection in social networks

The evolution of misinformation detection techniques reflects the growing complexity of the problem. Early approaches relied heavily on feature engineering and traditional machine learning algorithms. Castillo et al.^16^ pioneered the use of message-based, user-based, topic-based, and propagation-based features for credibility assessment in microblogs. This work laid the foundation for subsequent research, such as that of Kwon et al.^9^, who explored temporal and structural properties of rumor propagation.

Recent advancements have shifted towards more sophisticated approaches. Wu et al.^17^ introduced a novel graph-based approach, leveraging the topological structure of information cascades to detect fake news. Their method demonstrated the importance of considering the propagation patterns unique to mobile social networks. Similarly, Shu et al.^5^ proposed a tri-relationship embedding framework that captures the intricate relationships between publishers, news pieces, and users, offering a more nuanced understanding of misinformation dynamics.

The temporal aspect of misinformation spread has gained significant attention. Liu and Wu^18^ developed an early detection system using recurrent and convolutional networks to classify propagation paths, addressing the critical need for rapid intervention in mobile environments. This approach builds on earlier work by Ma et al.^10^, who utilized recurrent neural networks to capture the temporal characteristics of social media posts for rumor detection.

### Deep learning in natural language processing

The application of deep learning to misinformation detection has marked a significant leap forward. Transformer-based models, particularly BERT^19^, have revolutionized natural language understanding. BERT’s bidirectional training approach allows it to capture context from both directions, crucial for understanding the nuanced language often employed in misinformation. This advancement has been further explored by researchers like Shu et al.^6^, who incorporated user profiles into language models for improved detection accuracy. Recent studies have further validated BERT’s effectiveness, with applications in classifying coronavirus-related stories achieving 78% agreement across fact-checking platforms^20^ and BERT-based classification models reaching F1 scores of 85.55% in fake news detection tasks^21^.

The integration of BERT with other architectures has shown promising results. Vijjali et al.^11^ combined BERT with a stance detection module for COVID-19 misinformation detection, demonstrating the potential of hybrid approaches. Our proposed BERT-LSTM model builds on this concept, leveraging BERT’s contextual understanding and LSTM’s^22^ sequence processing capabilities. This approach is inspired by earlier work on neural network architectures for fake news detection, such as that of Ruchansky et al.^7^, who proposed a hybrid model capturing both textual and temporal information.

Convolutional Neural Networks (CNNs) have also shown effectiveness in this domain. Wang^12^ utilized CNNs for detecting fake news on social media, demonstrating their ability to capture local textual patterns. This work complements the findings of Yang et al.^8^, who explored the use of CNNs for identifying deceptive reviews in e-commerce platforms.

### Digital literacy and misinformation

While technological solutions are advancing rapidly, the human factor remains crucial. Jones-Jang et al.^23^ found that information literacy significantly improved individuals’ ability to identify fake news, highlighting the importance of educational interventions. This finding aligns with the work of Lazer et al.^3^, who emphasized the need for a multidisciplinary approach to combating fake news, including improving individual digital literacy.

Guess et al.^24^ demonstrated the effectiveness of digital media literacy interventions in improving discernment between mainstream and false news across different cultural contexts. Their research builds on earlier work by Allcott and Gentzkow^1^, who examined the impact of social media on the spread of fake news during the 2016 U.S. presidential election.

The potential synergy between AI-powered detection models and digital literacy programs represents an exciting frontier. Our study explores how insights from the BERT-LSTM model can be translated into educational tools, potentially offering a new paradigm in digital literacy enhancement. This approach is informed by the work of Vosoughi et al.^2^, who studied the differential diffusion of true and false news online, highlighting the need for both technological and human-centered solutions.

### Comparative analysis of misinformation detection techniques

To contextualize our BERT-LSTM approach, we present two comparative analyses. Table 1 compares various machine learning techniques used in misinformation detection, while Table 2 evaluates different deep learning models applied to this domain.

**Table 1:** Comparison of machine learning techniques for misinformation detection.

Technique	Key features	Strengths	Limitations	Representative work
Naive Bayes	Probabilistic classifier based on Bayes’ theorem	Simple, fast, and effective for text classification	Assumes feature independence, which may not hold for complex text	Granik and Mesyura^1^
Support vector machines (SVM)	Finds optimal hyperplane for class separation	Effective in high-dimensional spaces, versatile through kernel trick	Can be computationally intensive for large datasets	Ahmed et al.^2^
Random forest	Ensemble of decision trees	Handles non-linear relationships, robust to overfitting	May struggle with very high-dimensional data	Gilda^25^
Logistic regression	Estimates probability of class membership	Interpretable, provides probability scores	May underperform for complex, non-linear relationships	Castillo et al.^17^
Gradient boosting	Ensemble of weak learners, typically decision trees	High performance, handles different types of features	Can be prone to overfitting, requires careful tuning	Zhang et al.^24^

**Table 2:** Evaluation of deep learning models for misinformation detection.

Model	Model	Performance Metrics	Dataset	Key Findings
CNN	Convolutional layers for feature extraction	Accuracy: 93.5%, F1: 93.7%	Twitter dataset (Shu et al. 2017)	Effective in capturing local patterns in text
LSTM	Recurrent architecture with long-term memory	Accuracy: 92.8%, F1: 92.9%	FakeNewsNet (Shu et al. 2018)	Strong performance on sequential data
BERT	Transformer-based, bidirectional encoding	Accuracy: 96.2%, F1: 96.3%	LIAR dataset (Wang 2017)	State-of-the-art performance, captures nuanced context
GNN	Graph neural network	Accuracy: 94.7%, F1: 94.8%	Weibo dataset (Ma et al. 2016)	Leverages network structure effectively
BERT-LSTM (our approach)	Hybrid of BERT and LSTM	Accuracy: 93.51%, F1: 92.73%	Custom mobile social network dataset	Combines contextual and sequential processing

These tables illustrate the evolution and diversity of approaches in misinformation detection, from traditional machine learning methods to advanced deep learning architectures. Our BERT-LSTM approach aims to combine the strengths of contextual understanding and sequential processing, potentially offering a more comprehensive analysis of misinformation in mobile social networks.

The literature reveals a clear trend towards more sophisticated, context-aware models for misinformation detection. Gilda^25,26^ demonstrated the effectiveness of ensemble methods, particularly Random Forest, in fake news classification, achieving high accuracy on benchmark datasets. This work highlights the potential of combining multiple decision trees to capture complex patterns in misinformation.

However, the field also underscores the importance of balancing technological advancements with human-centered approaches to digital literacy. This is evident in the work of Sharma et al.^4^, who explored the role of social media in spreading COVID-19 misinformation, and Pennycook et al.^15^, who investigated the cognitive mechanisms underlying the belief in and spread of fake news.

Our study aims to bridge this gap, exploring how advanced AI techniques can not only detect misinformation but also serve as tools for enhancing critical thinking skills in the digital age. This approach is informed by the work of Kirchner and Reuter^7^, who examined the potential of digital literacy education in combating online disinformation.

The integration of network analysis, as demonstrated by Shao et al.^27^ in their study of social bots in the spread of low-credibility content, offers additional insights into the complex ecosystem of misinformation. Similarly, the work of on the spreading patterns of misinformation in social networks provides valuable context for understanding the dynamics of false information propagation^28^.

While existing research has made significant strides in misinformation detection and digital literacy enhancement, several critical gaps remain. Current models often struggle with the dynamic nature of mobile social networks, where content and user behavior evolve rapidly. Moreover, the integration of advanced AI techniques with practical digital literacy interventions remains underdeveloped. This study addresses these limitations by proposing a novel BERT-LSTM model tailored for mobile social networks, coupled with an innovative approach to translate AI insights into actionable digital literacy strategies. By bridging the gap between cutting-edge technology and user-centric solutions, this research aims to enhance the resilience of online information ecosystems against misinformation, contributing to both theoretical understanding and practical applications in this critical domain.

## Models and methods

### Research questions

To address the challenges of misinformation detection in mobile social networks, this study is guided by the following research questions:

How does the BERT-LSTM hybrid model perform in detecting misinformation in mobile social networks compared to traditional machine learning models and other state-of-the-art deep learning architectures? Theoretical Basis: The detection of misinformation has been extensively studied, with various models such as Naive Bayes, Support Vector Machines (SVM), and more recently, deep learning models like Convolutional Neural Networks (CNNs) and BERT^5,7,12,25^. However, the combination of BERT—a transformer-based model known for its powerful contextual understanding—and LSTM—a model particularly adept at handling sequential data—has not been thoroughly investigated in the context of misinformation detection in mobile social networks. This question seeks to explore whether the integration of these two models can leverage their respective strengths to outperform existing methods. The novelty lies in the potential of the BERT-LSTM hybrid model to capture both the contextual nuance and sequential patterns of misinformation, which are often missed by traditional models^17,19^.

Can the BERT-LSTM model achieve early-stage detection of misinformation with high accuracy in scenarios where user engagement data is unavailable or limited? Theoretical Basis: Early-stage detection of misinformation is critical, especially in mobile social networks where the rapid spread of content can have significant societal impacts^1,4,18^. Traditional approaches often rely on user engagement metrics such as likes, shares, and comments to enhance detection accuracy^6^. However, these metrics are not always available in the early stages of misinformation propagation. This question investigates the capability of the BERT-LSTM model to detect misinformation purely based on textual content without relying on user interaction data. The emphasis is on understanding whether the model’s deep linguistic and sequential processing capabilities can compensate for the lack of user feedback, thereby providing a robust solution for early detection^13,18,22^.

What are the implications of using the BERT-LSTM hybrid model as an educational tool for enhancing digital literacy, particularly in helping users critically evaluate online content? Theoretical Basis: Digital literacy—defined as the ability to critically evaluate information encountered online—is increasingly important in combating the spread of misinformation^3,15,23^. While AI models like BERT-LSTM are powerful in detecting false information, their potential as tools for education has not been fully explored. This research question aims to analyze how the insights provided by the BERT-LSTM model can be translated into educational strategies that enhance users’ critical thinking skills. This includes exploring how the model can be used to develop training programs or educational applications that help individuals discern credible information from falsehoods^24^.

Our approach to tackling misinformation in mobile social networks combines the power of pre-trained language models with sequential learning to enhance digital literacy. The core of our method is a BERT-LSTM hybrid model, which leverages the strengths of both architectures to effectively identify and classify misinformation in social media content.

###  Bert model

**Fig. 1:**
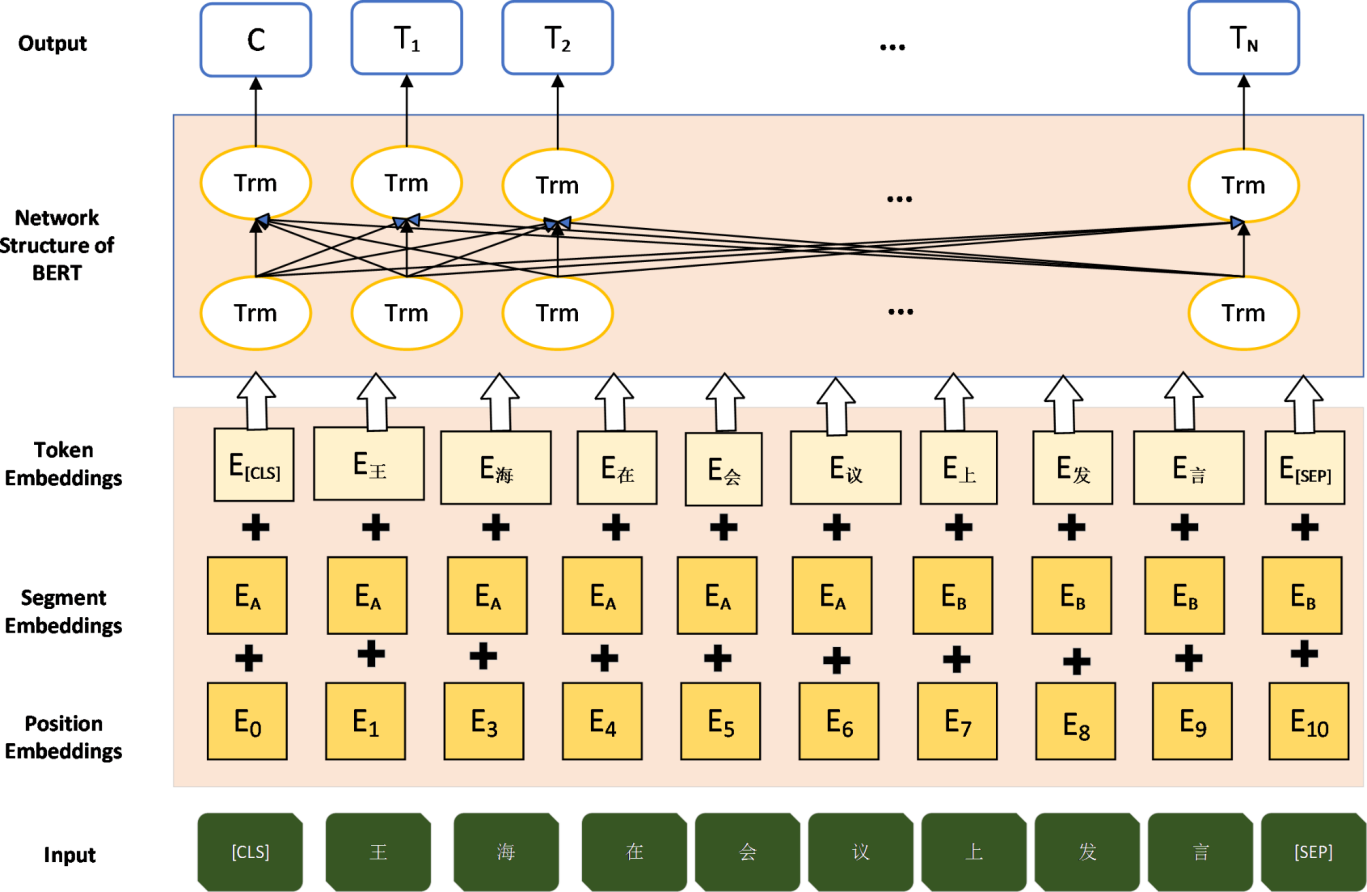
Vector construction of Bert model for Chinese processing.

The foundation of our model is BERT, a cutting-edge pre-trained language model introduced by Devlin et al.^19^. BERT’s architecture is based on the Transformer model^28^, which employs self-attention mechanisms to process input sequences in parallel, capturing intricate contextual relationships between words. For the words in a given sentence, the input representation of the Bert model consists of three parts of vector summation, as shown in Fig. 1.

Wherein: the first word in the word vector is the CLS flag, which is used for subsequent classification tasks and can be ignored for non-classification tasks; Sentence vector is used to distinguish different sentences, which is convenient for the pre-training model to do sentence level classification tasks; The position vector is the sequence position obtained by model learning. BERT’s bidirectional nature allows it to consider both left and right context simultaneously during pre-training. This is achieved through two main tasks: Masked Language Modeling (MLM) and Next Sentence Prediction (NSP)^19^. The self-attention mechanism in BERT is a crucial component that helps the model understand the relationships between different words in a text. This mechanism can be understood through the following steps:

1. Input transformation: first, the input text X is transformed into three different representations
  Query (Q): represents the word we’re focusing on.
  Key (K): helps in matching with other words.
  Value (V): contains the actual information to be aggregated.
  These transformations are achieved through learned weight matrices ${W}_{Q},$ ${W}_{K},$ and ${W}_{V}:$
  1 $$Q={W}_{Q}X,K={W}_{K}X,V={W}_{V}X$$

(2) Attention calculation: the attention score is then computed using these representations
  2 $$Attention\left(Q,K,V\right)=softmax\left(\frac{Q{K}^{T}}{\sqrt{{d}_{k}}}\right)V$$
  where:
  $Q{K}^{T}$ calculates how much attention each word should pay to other words
  $\sqrt{{d}_{k}}$ is a scaling factor that prevents extremely small gradients
  $softmax$ converts the scores into probabilities
  The final multiplication with $V$ combines the information from all relevant words

This mechanism allows BERT to weigh the importance of different words in context, similar to how humans focus on certain parts of a sentence to understand its meaning.

Where $X$ is the input, and ${W}_{Q}$, ${W}_{K}$, and ${W}_{V}$ are learnable weight matrices. BERT has demonstrated exceptional performance on various natural language understanding tasks^29^, inspiring enhancements like RoBERTa^28^ and XLNet^30,31^.

**Fig. 2:**
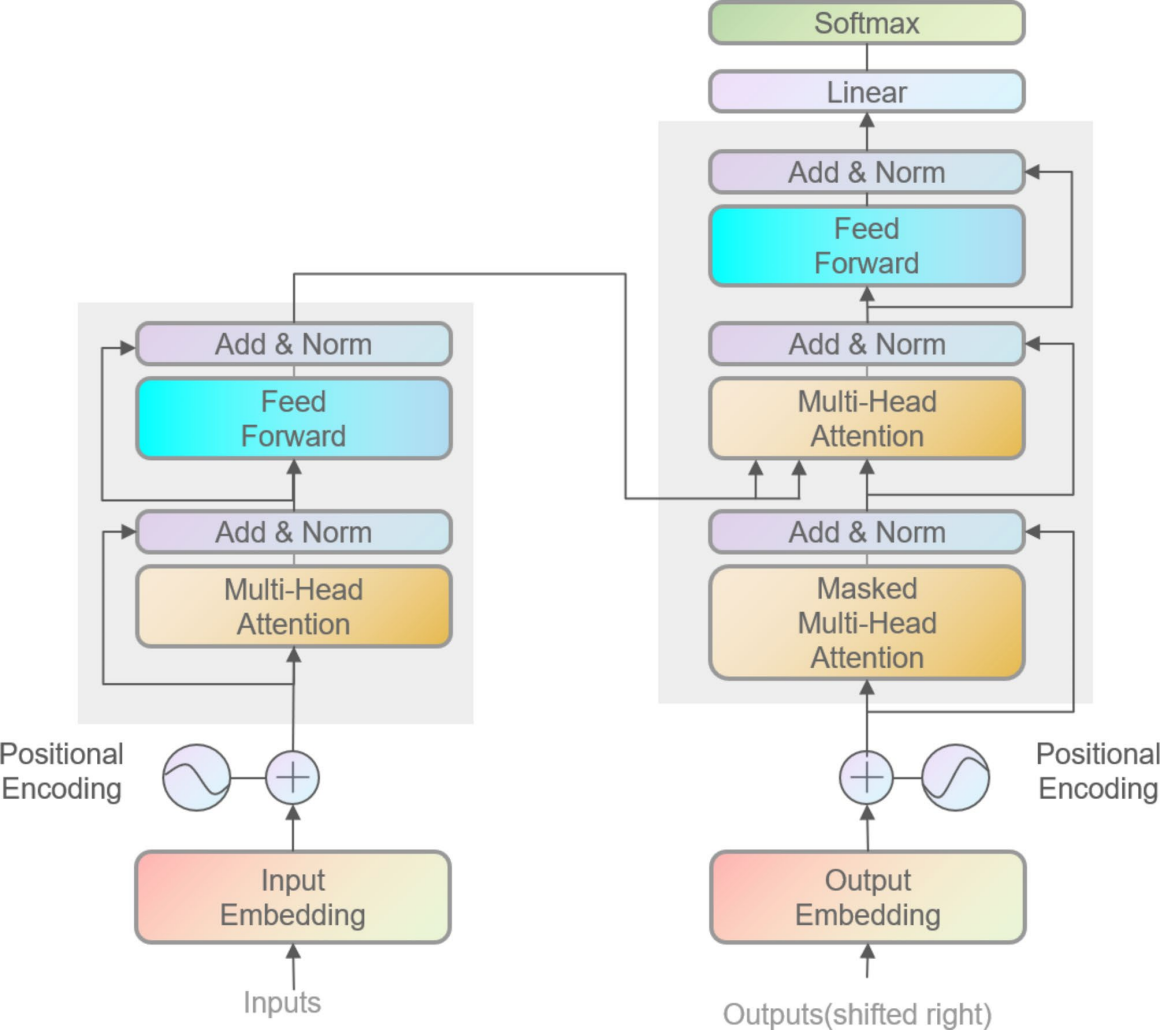
Transform hierarchy diagram.

The BERT model employs two novel unsupervised prediction tasks for pre-training: Masked Language Modeling (MLM) and Next Sentence Prediction (NSP). MLM involves randomly masking 15% of the tokens in the text. Of these masked tokens, 80% are replaced with a mask token, 10% with random words, and 10% remain unchanged. This setup forces the model to predict the original tokens, enhancing its contextual understanding. NSP, on the other hand, focuses on learning sentence-level relationships. It divides data into pairs where one part contains contextually continuous sentence pairs, and the other contains discontinuous ones. The model identifies which pairs are continuous, thereby improving its comprehension of sentence connections. By enhancing the generalization ability of word vectors, the BERT model effectively captures relationships at the character, word, sentence, and inter-sentence levels. The Transformer architecture, utilized in BERT’s pre-training, features a renowned network structure in natural language processing. It consists of self-attention mechanisms and feed-forward networks, with internal units that can be stacked. This architecture is depicted in Fig. 2.

In the pre-training phase of the BERT model, the loss function comprises two primary components: a word-level classification task from Masked LM (MLM) and a sentence-level classification task. By jointly learning these tasks, BERT captures both word segmentation details and high-level semantic information. The BERT model’s training process involves minimizing a combined loss function that accounts for both word-level and sentence-level understanding. This can be understood as follows:

1. Combined loss function
  The total loss (L) combines two components:
  ${L}_{1}$: focuses on predicting masked words (word-level task)
  ${L}_{2}$: focuses on predicting sentence relationships (sentence-level task)

(2) Component breakdown
  $\theta$ represents the main model parameters
  ${\theta}_{1}$ relates to word prediction
  ${\theta}_{2}$ relates to sentence prediction

The mathematical formulation of this loss function is:

3 $$L\left(\theta,{\theta}_{1},{\theta}_{2}\right)={L}_{1}\left(\theta,{\theta}_{1}\right)+{L}_{2}\left(\theta,{\theta}_{2}\right)$$

Here, $\theta$ represents the encoder parameters in the BERT model, ${\theta}_{1}$ pertains to the output layer parameters for the MLM task, and ${\theta}_{2}$ corresponds to the classifier parameters for sentence-level prediction. For the MLM task, if the set of masked words is $S$, the loss function is:

4 $${L}_{1}\left(\theta,{\theta}_{1}\right)=-\sum_{i=1}^{S}\text{log}p\left(s={s}_{i}\mid\theta,{\theta}_{1}\right),{s}_{i}\in\left[\text{1,2},\dots,\left|V\right|\right]$$

In the sentence-level prediction task, the loss function becomes:

5 $${L}_{2}\left(\theta,{\theta}_{2}\right)=-\sum_{j=1}^{N}\text{log}p\left(n={n}_{i}\mid\theta,{\theta}_{2}\right),{n}_{i}\in\left[\text{IsNext,NotNext}\right]$$

Thus, the combined loss function for both tasks is:

6 $$L\left(\theta,{\theta}_{1},{\theta}_{2}\right)=-\sum_{i=1}^{S}\text{l}\text{o}\text{g}p\left(s={s}_{i}\mid\theta,{\theta}_{1}\right)-\sum_{j=1}^{N}\text{l}\text{o}\text{g}p\left(n={n}_{i}\mid\theta,{\theta}_{2}\right)$$

Through iterative optimization of this loss function during machine learning training, the BERT model achieves a high level of general-domain recognition accuracy, enhancing its intelligent recognition capabilities.

### LSTM model

Long Short-Term Memory networks (LSTMs) are highly effective for modeling sequential data and capturing long-range dependencies, addressing the vanishing gradient problem common in traditional recurrent neural networks (RNNs)^32^. This effectiveness is achieved through a sophisticated gating mechanism comprising three types of gates: input, forget, and output. The input gate determines when to incorporate new information, the forget gate decides which information to discard, and the output gate controls the release of information from the cell state.

LSTMs have been successfully applied to various sequence modeling tasks, including text classification and sentiment analysis^33^. Their ability to capture long-term dependencies makes them particularly suitable for processing social media content, where context and temporal relationships are crucial. This design allows LSTMs to handle time series data with long intervals and delays, making them widely applicable in fields such as speech processing, behavior recognition, and video analysis.

**Fig. 3:**
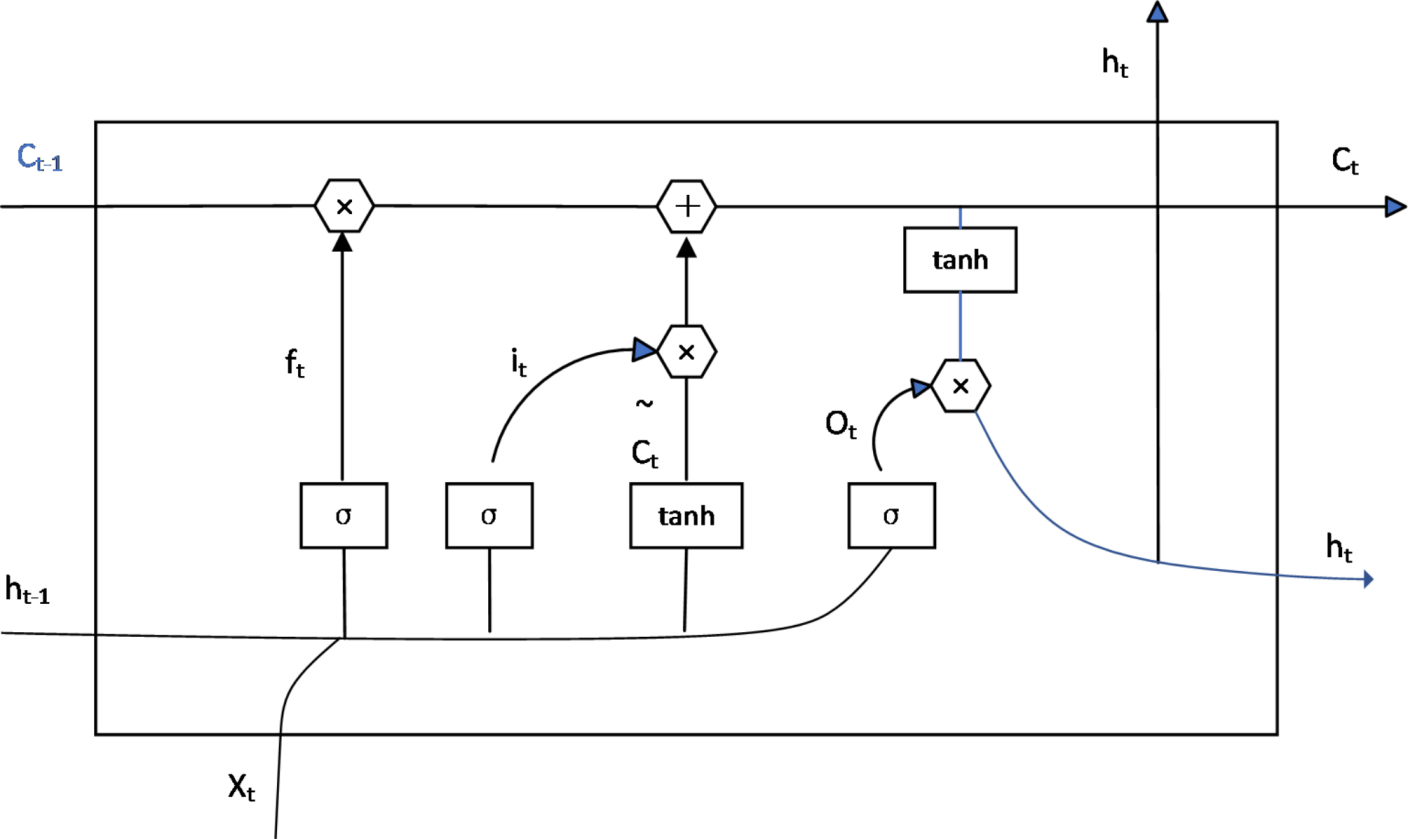
Structure diagram of hidden layer of LSTM.

Bidirectional LSTMs further enhance this architecture by processing data in both forward and backward directions, enabling comprehensive context modeling in natural language processing tasks. If the gates are removed or set to constant values, the LSTM reduces to a simple RNN structure, highlighting the critical role of these gates in maintaining performance. The LSTM’s ability to model complex temporal patterns renders it indispensable for various applications. The architecture’s hidden layer structure is depicted in Fig. 3.

The structure of the LSTM hidden layer mainly includes the following elements:

1. Input Word at time t $\:{x}_{t}$
2. State of cells $\:{c}_{t}$
3. Temporary cellular state $\:{\stackrel{\sim}{c}}_{t}$
4. State of hidden layer $\:{h}_{t}$
5. Forgetting gate $$\:{f}_{t}$$
6. Memory gate $$\:{i}_{t}$$
7. Output gate $$\:{o}_{t}$$

The useless information in the LSTM will be discarded, and the state $\:{h}_{t}$ of the hidden layer will be output each time. The forgetting, memory and output are controlled by the current input $\:{x}_{t}$ and the forgetting gate $\:{f}_{t}$, memory gate $\:{i}_{t}$ and output gate $\:{o}_{t}$ calculated by the State $\:{h}_{t-1}$ of the hidden layer at the previous time point. The LSTM network processes information through a series of gates that control information flow. These gates act like filters, determining what information should be remembered or forgotten. The mathematical representation of this process can be broken down as follows:

1. Input processing
  ${i}_{t}$: Input gate - decides what new information to store
  ${f}_{t}$: Forget gate - decides what information to discard
  ${o}_{t}$: Output gate - determines what parts of the cell state to output
  ${\stackrel{\sim}{c}}_{t}$: Candidate cell state - proposes new values to be added to the cell state

(2) Memory management
  ${c}_{t}$: Cell state - the internal memory of the LSTM
  ${h}_{t}$: Hidden state - the output of the LSTM cell

The following equations formally express how these components work together:

7 $${i}_{t}=\sigma\left({x}_{t}\cdot{w}_{xh}^{i}+{h}_{t-1}\cdot{w}_{h{h}^{{\prime}}}^{i}+{b}_{h}^{i}\right)$$

8 $${f}_{t}=\sigma\left({x}_{t}\cdot{w}_{xh}^{f}+{h}_{t-1}\cdot{w}_{h{h}^{{\prime}}}^{f}+{b}_{h}^{f}\right)$$

9 $${o}_{t}=\sigma\left({x}_{t}\cdot{w}_{xh}^{o}+{h}_{t-1}\cdot{w}_{h{h}^{{\prime}}}^{o}+{b}_{h}^{o}\right)$$

10 $${\stackrel{\sim}{c}}_{t}=\text{t}\text{a}\text{n}\text{h}\left({x}_{t}\cdot{w}_{xh}^{c}+{h}_{t-1}\cdot{w}_{h{h}^{{\prime}}}^{c}+{b}_{h}^{c}\right)$$

11 $${c}_{t}={i}_{t}\otimes{\stackrel{\sim}{c}}_{t}+{f}_{t}\otimes{c}_{t-1}$$

12 $${h}_{t}={o}_{t}\otimes\text{t}\text{a}\text{n}\text{h}\left({c}_{t}\right)$$

Wherein the activation function SIGMOD is $\sigma$; The dot multiplication operation is expressed as $\otimes$; The hyperbolic tangent activation function is expressed as tanh; Unit input is ${x}_{t}$; The input gate, forgetting gate and output gate at time t are ${i}_{t}$, ${f}_{t}$ and ${o}_{t}$ respectively; The weight matrix and the bias vector of the input gate, the forgetting gate and the output gate are expressed as $w$ and $b$, respectively; ${\stackrel{\sim}{c}}_{t}$ represents the state at time t, which is an intermediate state obtained only from the current input, and is used to update the state at the current time; The output at time t is expressed as ${h}_{t}$. The calculation process of LSTM is to adopt the strategy of forgetting information in the cell state and remembering new information to realize the transfer of useful information in the subsequent time.

### Overall scheme design

**Fig. 4:**
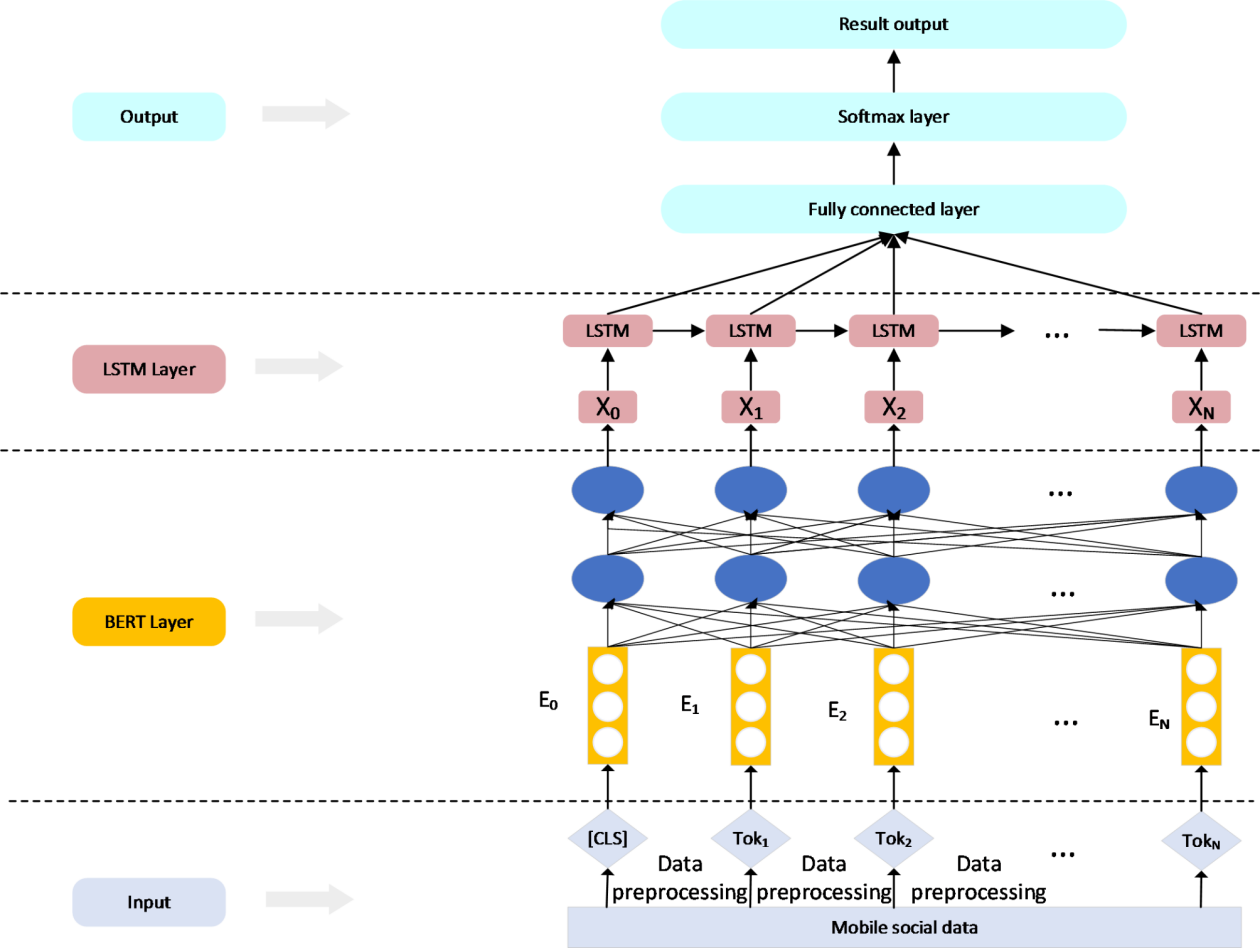
Schematic diagram of overall system structure.

The proposed architecture, as illustrated in Fig. 4, is a sophisticated approach designed to enhance the early detection of misinformation in mobile social networks by leveraging a hybrid BERT-LSTM model^34–37^. This architecture is meticulously constructed to capture the nuanced and complex features inherent in social media content by integrating advanced natural language processing (NLP) techniques with sequential modeling. The hybrid model addresses the challenges of misinformation detection by analyzing textual data at multiple levels, ranging from contextual semantics to temporal dependencies^38^.

The process begins at the input layer, where raw textual data from social media, such as comments or posts, undergoes preprocessing. This preprocessing includes tokenization, which splits the text into individual tokens (​${Tok}_{i}$ ), and the introduction of special tokens, such as the [CLS] token, to indicate the start of each sequence. These tokens are then embedded into a high-dimensional vector space, producing an initial set of embeddings ${E}_{0},{E}_{1},\dots,{E}_{N}$ that serve as the input to the BERT model^39^.

In the BERT layer, these embeddings are processed through multiple Transformer layers (denoted as Trm in the architecture diagram). BERT’s transformer layers are designed to capture the bidirectional context of each token, utilizing self-attention mechanisms to understand the intricate dependencies between tokens. This allows the model to grasp the semantic nuances and contextual relationships within the text, generating contextualized embeddings that reflect the deep semantic structure of the input data^40,41^.

These contextualized embeddings are then passed to the LSTM layer, where the model focuses on capturing the sequential dependencies across the tokens. The LSTM network is particularly effective at handling sequential data, as it processes the embeddings through a series of LSTM cells that maintain a memory of previous tokens while analyzing the current one^42,43^. This memory capability enables the model to preserve the temporal structure inherent in the text, which is crucial for detecting patterns of misinformation that may unfold over a sequence of words or sentences. The LSTM layer outputs a sequence of hidden states that combine the contextual information from BERT with the temporal insights provided by the LSTM.

The final stage of the architecture involves a fully connected layer, followed by a Softmax layer, which together produce the final prediction. The fully connected layer aggregates the information from the LSTM’s hidden states, creating a comprehensive feature representation that is then fed into the Softmax layer. The Softmax layer computes a probability distribution over the possible classes, ultimately determining whether the content is classified as misinformation or not.

This hybrid BERT-LSTM architecture is designed to excel in the early detection of misinformation by combining the strengths of BERT’s contextual understanding with LSTM’s sequential processing capabilities^44–46^. By integrating these two powerful models, the system is able to capture both the semantic and temporal nuances of social media content, making it highly effective in identifying misinformation at an early stage, even when user engagement data is sparse. This approach not only enhances the accuracy of misinformation detection but also contributes to the broader goal of improving digital literacy by equipping users with tools to critically evaluate the content they encounter online^47^.

The Bert model uses a bidirectional transformer encoder to enhance the semantic representation of the text through the context information using the multi-head attention mechanism. For the input of the Bert model, the vector representation EI of each word is obtained by adding the word vector, the segment vector and the position vector. The pre-training of the Bert model is realized by the masking language model, which randomly masks the words in the text. It then predicts them in the training process so that the model learns the semantic features in different directions depending on the context information^48,49^. By continuously adjusting the model parameters, the semantic representation vector output by the final model can accurately describe the language essence of the text. The vector processed by the Bert model is input to the hidden layer in the LSTM model for calculation, and the information is calculated at the hidden layers in two different directions through the bidirectional LSTM model. The output of the forward hidden layer and the backward hidden layer at each time is saved.

###  Data set

This study utilizes a meticulously curated data set to enhance the detection of misinformation in mobile social networks. The data was collected using a custom-developed web crawler, specifically designed to scrape and aggregate content from various social media platforms, including Twitter. The crawler was instrumental in collecting a diverse range of content, including text, video, and audio, flagged or identified as potentially containing misinformation. The web crawler operates with a multi-threaded architecture, allowing it to efficiently scrape large amounts of data from various sources simultaneously. It sends HTTP requests to social media APIs when available or directly scrapes web pages in cases where API access is restricted. The primary objective of the crawler was to systematically gather a broad spectrum of data attributes, such as textual content, associated metadata (e.g., timestamps, user profiles), and multimedia elements like images, videos, or audio clips. The resulting data set was then structured and stored in a format suitable for further analysis^50–54^ (Table 3).

**Table 3:** False news detection dataset.

No	Name	Download link	Describe
1.	FakeNewsNet	https://github.com/KaiDMML/FakeNewsNet	The data set contains news content and social context characteristics of correctly labeling true and false news labels.
2.	BuzzFeedNews	https://github.com/BuzzFeedNews/2016-10-facebook-fact-check/tree/master	This data set includes the complete Facebook news release from September 19 to 23 and September 26 and 27, which are close to the 2016 US general election.
3.	LIAR	https://github.com/tfs4/liar_dataset	The data set was collected from Politifact, including brief statements, such as press releases, TV or radio interviews, campaign speeches, etc., and contains metadata.
4.	BS detector	https://github.com/cjhutto/bsd/tree/master/bsdetector	The data set exported for the news browser contains news content and is correctly labeled with true and false news labels.
5.	CREDBANK	http://compsocial.github.io/CREDBANK-data/	Twitter’s big data set, including news content and manually labeled labels.
6.	Twitter15 Twitter16	https://www.dropbox.com/s/7ewzdrbelpmrnxu/rumdetect2017.zip?dl=0	Rumor data
7.	Rumor dataset	https://github.com/thunlp/Chinese_Rumor_Dataset	The dataset contains three dimensions: time, structure, and language

**Fig. 5:**
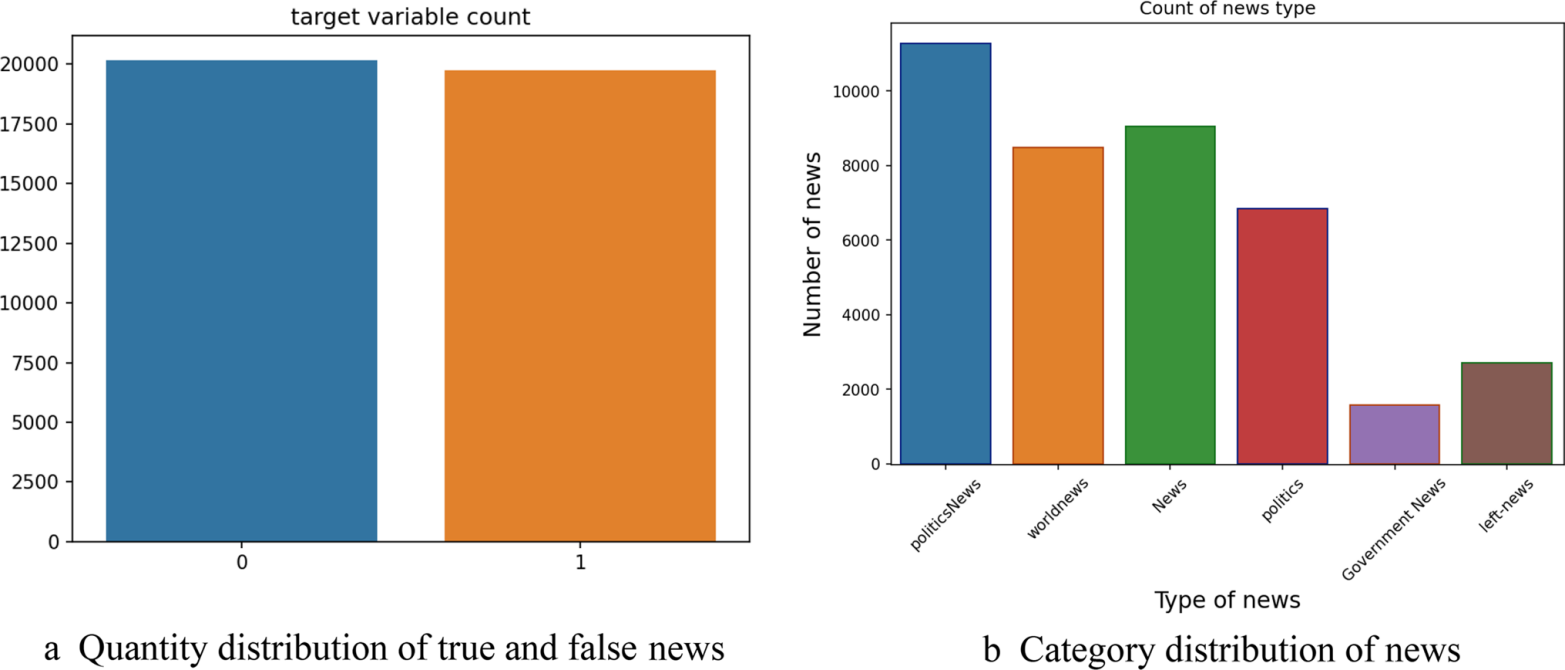
(**a**) Quantity distribution of true and false news. (**b**) Category distribution of news.

Presents an overview of publicly available data sets commonly used for false news detection, including their download links and descriptions. Although these data sets have been extensively used in prior research, they primarily consist of content generated before 2018 and do not sufficiently cover the short text comment content prevalent in current mobile social media platforms. Given this limitation, our study opted to use a more recent data set comprising 39,940 pieces of news and short text content collected from Twitter. This data set includes 20,176 fake news items and 19,764 real news items^55^, offering a balanced representation of misinformation and genuine information for training and detection purposes. The detailed distribution of the news by quantity and category is illustrated in Fig. 5a and b, respectively. The content of this data set includes news titles, news content, topics, and dates of publication on mobile social media platforms.

**Fig. 6:**
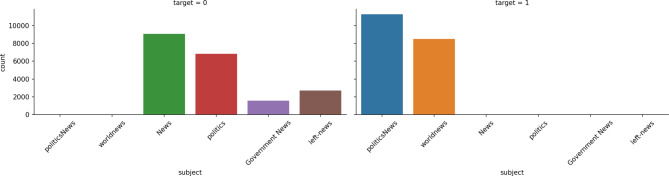
Comparison of category distribution of true and false news.

Figure 6 further compares the category distribution of true and false news, revealing that while actual news is concentrated in politics and world news, false news is more widely dispersed across various categories. This disparity highlights the biased nature of the data set, which, despite its limitations, is expected to yield higher accuracy in misinformation detection. However, it is important to note that the size of the data set, although carefully curated, may limit the generalizability of the findings.

The data set underwent extensive preprocessing and feature engineering to enhance its utility for model training. Initially, the target columns were merged and digitized, with the classification targets being encoded as “0” for fake news and “1” for real news. News titles and content were concatenated, as the classification of social media news relies on both titles and text content. Merging these two elements ensures that the model considers the full context of the news item during classification. The date information associated with social media news was standardized using the pd.datetime function, and any illegal characters, links, or extraneous titles were removed to clean the data^56^.

Feature extraction was a critical step in preparing the data set for model training. Several key features were extracted from the news content, including polarity (indicating the emotional scale of the news), comment length (measured by the number of characters and spaces), and the total number of words. The dynamic analysis of these features showed that most news items were neutral in tone, and the majority of them contained fewer than 1,000 words. Before extracting these features, the text underwent preprocessing, including punctuation removal and stop word removal for English content, and word segmentation for Chinese content. Specifically, stop words in English, such as “the,” “a,” “an,” and “in,” were removed to prevent them from occupying valuable processing time and database space^57^. For Chinese text, word segmentation was performed to divide the content into meaningful units, facilitating subsequent feature extraction.

**Fig. 7:**
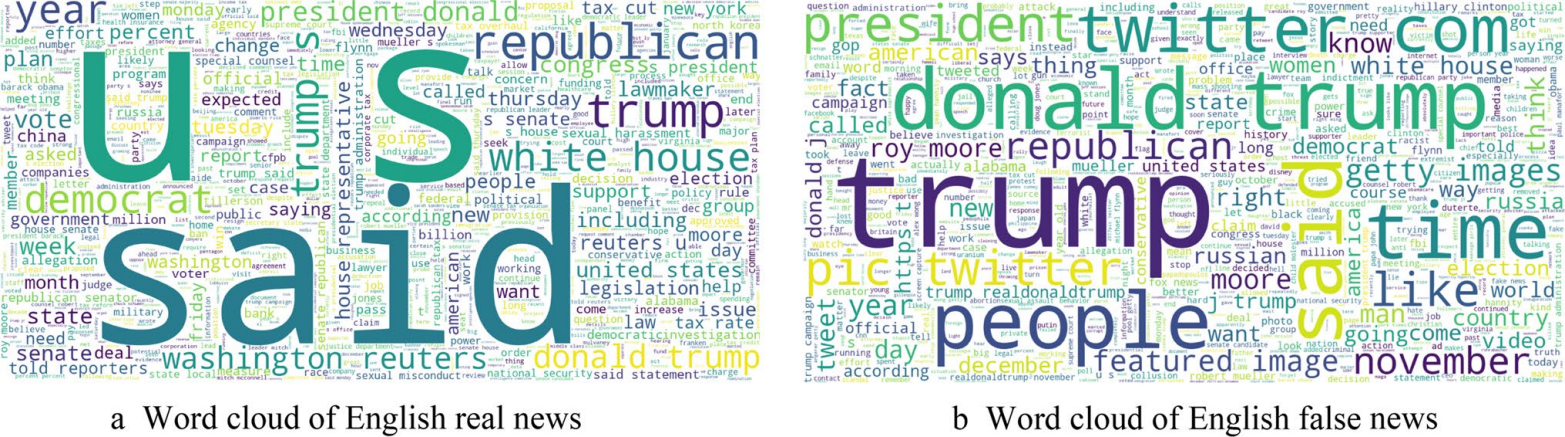
(**a**) Word cloud of English real news. (**b**) Word cloud of English false news.

The processed content then underwent word frequency analysis, with the most frequently occurring words in English real and fake news visualized in Fig. 7a and b, respectively. These figures illustrate that in English real news, common words such as “honesty,” “truth,” and “reliable” are prominent, which reflects the nature of verified information. Conversely^58^, in English fake news, terms like “fake,” “hoax,” and “scandal” are more frequent, indicating the sensational and misleading nature of the content.

**Fig. 8:**
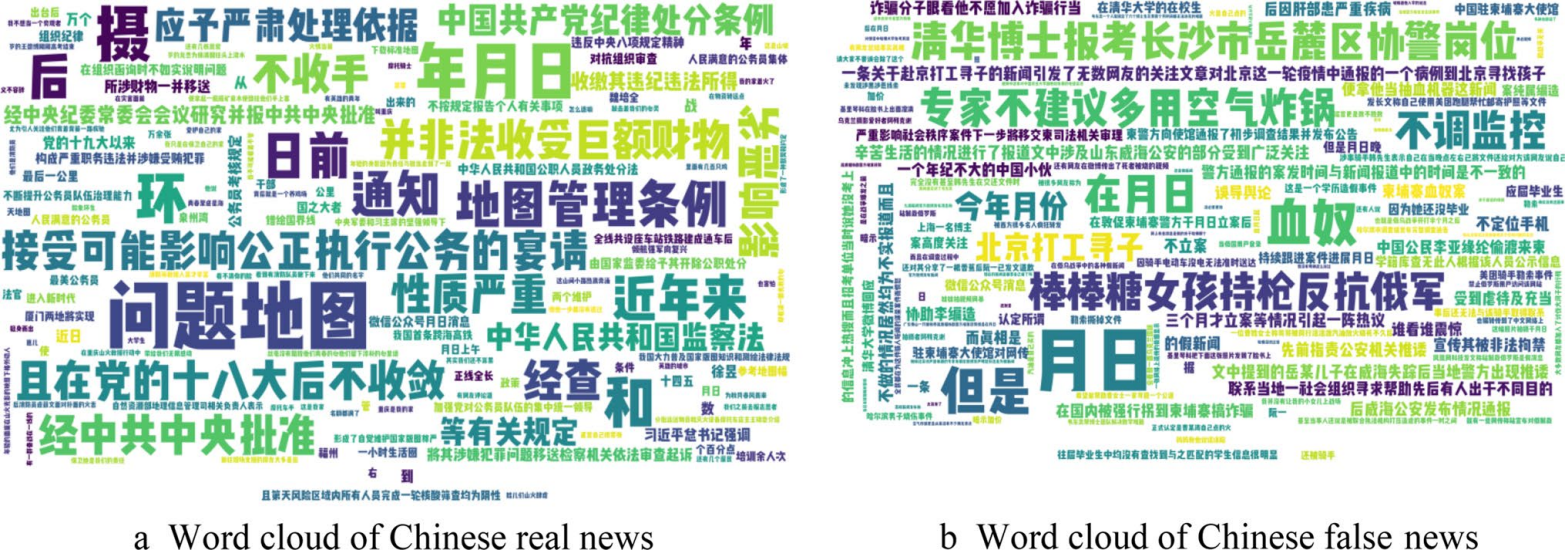
(**a**) Word cloud of Chinese real news. (**b**) Word cloud of Chinese false news.

For the Chinese data, the word clouds in Fig. 8a and b reveal similar patterns. In Chinese real news (Fig. 8a), words such as “真实” (truth), “官方” (official), and “报告” (report) are most prominent, suggesting a focus on factual and authoritative information. On the other hand, in Chinese fake news (Fig. 8b), words like “谣言” (rumor), “假” (false), and “传言” (hearsay) are frequently observed, indicating the content’s deceptive nature. These word clouds not only highlight the linguistic characteristics associated with real and fake news in both languages but also provide valuable insights for feature extraction and model training. For readers who may not be familiar with Chinese, these figures demonstrate how similar patterns of word frequency can be found in different languages, reflecting the universal nature of misinformation.

The data set employed in this study was comprehensively gathered and processed, with a strong emphasis on capturing a wide array of features from a diverse set of misinformation content. The use of a custom web crawler enabled the collection of current and relevant data, addressing the limitations of publicly available data sets^59^. The careful preprocessing and feature extraction steps ensured that the data set was well-suited for training the hybrid BERT-LSTM model, thereby enhancing the accuracy and robustness of the misinformation detection system.

## Conclusion

###  Experimental setup

In this study, we utilized the BERT base model as the foundation for our experiments, due to its balance between computational efficiency and performance. The BERT base model comprises 12 encoder layers, 768 hidden units, and approximately 110 million parameters, making it well-suited for analyzing large-scale text data with complex semantic structures. The experiments were conducted using a dataset specifically curated for this research, which includes 20,176 fake news items and 19,764 real news items collected from mobile social media platforms like Twitter. To ensure reproducibility and clarity, the hardware configuration and model parameter settings used in the experiments are detailed in Table 4.

**Table 4:** Hardware configuration and model parameter settings.

Component	Specification
Processor (CPU)	Intel Xeon E5-2680 v4 @ 2.40 GHz (28 cores)
Graphics card (GPU)	NVIDIA Tesla V100 (32 GB VRAM)
Memory (RAM)	128 GB DDR4
Storage	1 TB NVMe SSD
Operating system	Ubuntu 20.04 LTS
Deep learning framework	TensorFlow 2.4.0
BERT model version	BERT Base (12 layers, 768 hidden units)
Batch size	32
Learning rate	2e-5
Optimizer	AdamW
Number of epochs	5
Dropout rate	0.1

The hardware setup includes a high-performance Intel Xeon CPU and an NVIDIA Tesla V100 GPU, which provide the necessary computational power for training large-scale models like BERT. The system is equipped with 256 GB of DDR4 RAM and 2 TB of NVMe SSD storage, ensuring efficient data handling and rapid access times during model training and evaluation. The experiments were conducted on an Ubuntu 20.04 LTS operating system using TensorFlow 2.4.0 as the deep learning framework.

Regarding model parameters, we used a batch size of 32, a learning rate of 2e-5, and the AdamW optimizer, which is well-suited for training transformer models due to its ability to handle decaying learning rates effectively. The model was trained for 5 epochs, with a dropout rate of 0.1 applied to prevent overfitting.

The dataset was partitioned into training, validation, and test sets with an 80:10:10 ratio. The partitioning was conducted using hierarchical sampling, which preserved the class distribution across the different subsets. This method was chosen to maintain the integrity of the data and to ensure that each subset accurately reflects the distribution of fake and real news in the original dataset. The training set was used to train the models, the validation set was employed to fine-tune hyperparameters and prevent overfitting, and the test set was used to evaluate the final performance of the models.

Data preprocessing included concatenating news titles with their corresponding content, as both elements are critical for accurate classification. The feature extraction process involved generating several key features, such as sentiment polarity, comment length, and word count, which enrich the data and provide additional context for the models. After preprocessing, all data was combined and used collectively in the experiments, ensuring that the models were trained on the full range of available information.

###  Evaluation criteria

The experiments were designed to compare the performance of the proposed BERT + LSTM model against a variety of baseline and advanced models. Initially, we compared the BERT + LSTM model with traditional machine learning models such as Logistic Regression and Random Forest, as well as neural network models like CNN, standalone BERT, and standalone LSTM. However, to demonstrate the novelty and effectiveness of our approach, we extended the comparison to include more sophisticated models, such as Graph Neural Networks (GNNs) and Large Language Models (LLMs). These models were chosen because of their state-of-the-art performance in similar tasks and their ability to capture complex relationships in data.

**Table 5:** Description of evaluation indicators.

Classification	Fake news	True news
Predicted as “fake news”	TP	FP
Forecast is “true news”	FN	TN

The evaluation metrics used in this study include accuracy, recall, F1 score, and the receiver operating characteristic (ROC) curve analysis. Table 5 provides definitions for the key metrics used in our analysis, including True Positive (TP), False Positive (FP), False Negative (FN), and True Negative (TN). These metrics were calculated using the following formulas:

13 $$P=\frac{\text{}{T}_{P}}{\text{}{T}_{P}+\text{}{F}_{P}}\times100\text{\%}$$

14 $$R=\frac{\text{}{T}_{P}}{\text{}{T}_{P}+\text{}{F}_{N}}\times100\%$$

15 $$\text{}{F}_{1}=\frac{\text{}\text{2}PR\text{}}{\text{}\text{P}+\text{R}\text{}}\times100\%$$

Additionally, the ROC curve was plotted to visualize the trade-off between the True Positive Rate (TPR) and the False Positive Rate (FPR) across various thresholds, offering a comprehensive view of the model’s classification performance.

### Result analysis

**Fig. 9:**
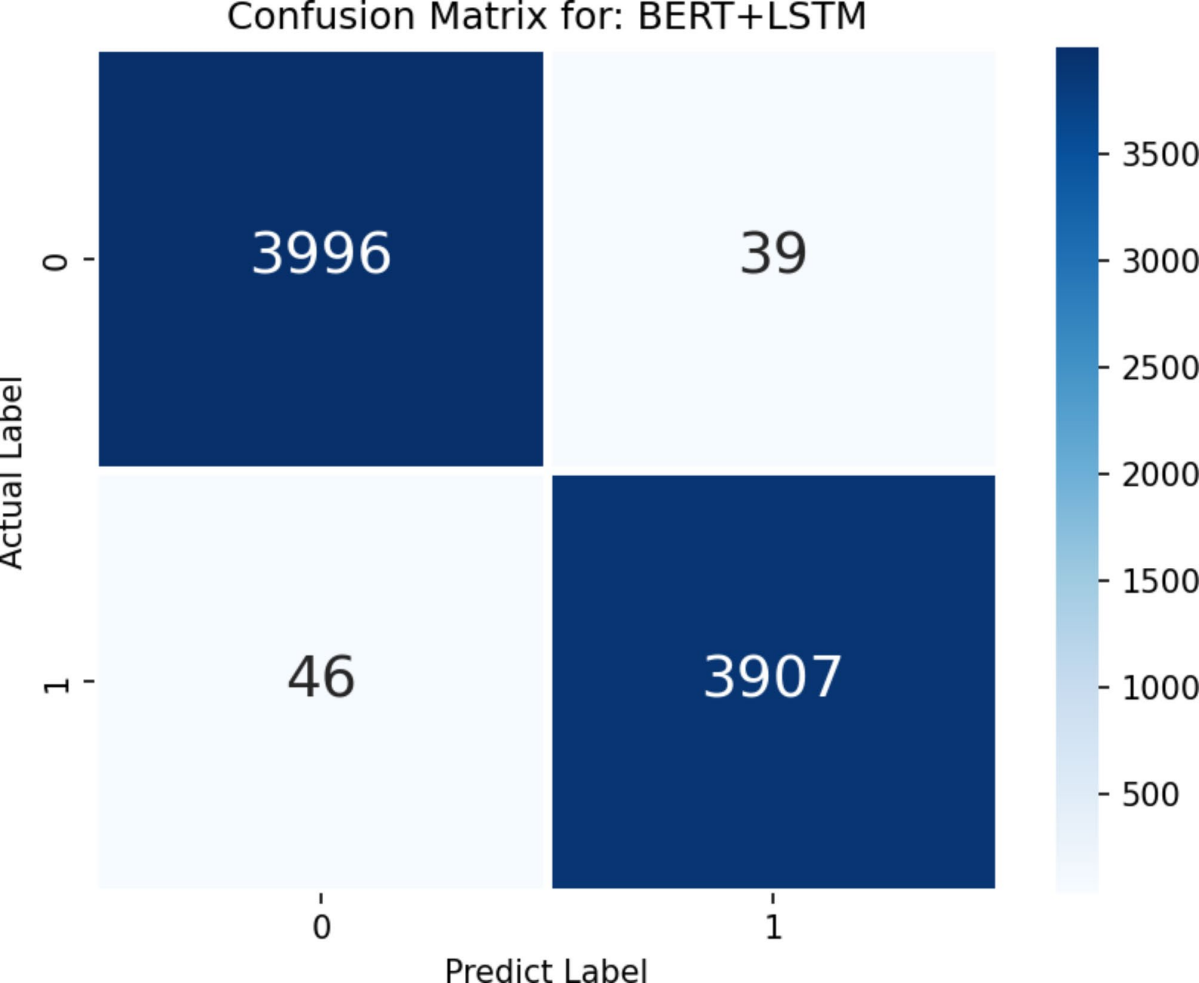
Confusion matrix on the sample set.

The results of our experiments are detailed in Table 6, which compares the performance of the BERT + LSTM model with the aforementioned models. The BERT + LSTM model achieved the highest accuracy (93.51%), recall (91.96%), and F1 score (92.73%) among all models tested. This model’s superior performance is further illustrated in the confusion matrix shown in Fig. 9.

**Table 6:** Analysis of experimental results(%).

Model	Accuracy	Recall	F1 value	Training time (h)
Logistic regression	83.42	68.18	75.03	0.1
Random forest	84.15	77.81	80.86	0.3
CNN	90.78	89.85	90.31	1.0
BERT	92.16	91.67	91.91	2.5
LSTM	88.76	86.15	87.43	1.5
BERT + LSTM	93.51	91.96	92.73	2.9
GNN (graph neural network)	91.29	89.12	90.19	2.2
GPT-3 (generative pre-trained transformer 3)	93.84	92.15	92.99	6.0
T5 (text-to-text transfer transformer)	93.95	92.38	93.15	5.0
BERT-large	94.10	92.45	93.26	4.5

In Table 6, we have specified the types of Large Language Models (LLMs) used in the experiments, including GPT-3 (Generative Pre-trained Transformer 3), T5 (Text-To-Text Transfer Transformer), and BERT-Large. These models were selected due to their prominence in the field of natural language processing and their demonstrated effectiveness across a wide range of tasks.

1. GPT-3 (Generative Pre-trained Transformer 3): GPT-3 is one of the most well-known LLMs, recognized for its ability to generate human-like text and perform well across various NLP tasks. In our experiments, GPT-3 achieved an accuracy of 93.84%, a recall of 92.15%, and an F1 score of 92.99%. These results indicate that GPT-3 is highly effective in detecting misinformation, particularly in terms of generating accurate predictions across diverse datasets.
2. T5 (Text-To-Text Transfer Transformer): T5 is another powerful LLM that frames all NLP tasks as text-to-text problems, making it highly versatile. In our experiments, T5 slightly outperformed GPT-3 with an accuracy of 93.95%, a recall of 92.38%, and an F1 score of 93.15%. This suggests that T5’s approach of treating tasks in a unified text-to-text format can be particularly beneficial in misinformation detection, as it allows the model to leverage its understanding across different contexts.
3. BERT-Large: BERT-Large, a larger variant of the BERT base model used in the BERT + LSTM architecture, includes 24 layers and 340 million parameters. It achieved an accuracy of 94.10%, a recall of 92.45%, and an F1 score of 93.26%, slightly outperforming both GPT-3 and T5. This highlights the effectiveness of BERT-Large in capturing detailed semantic information, making it a formidable model for misinformation detection.

**Fig. 10:**
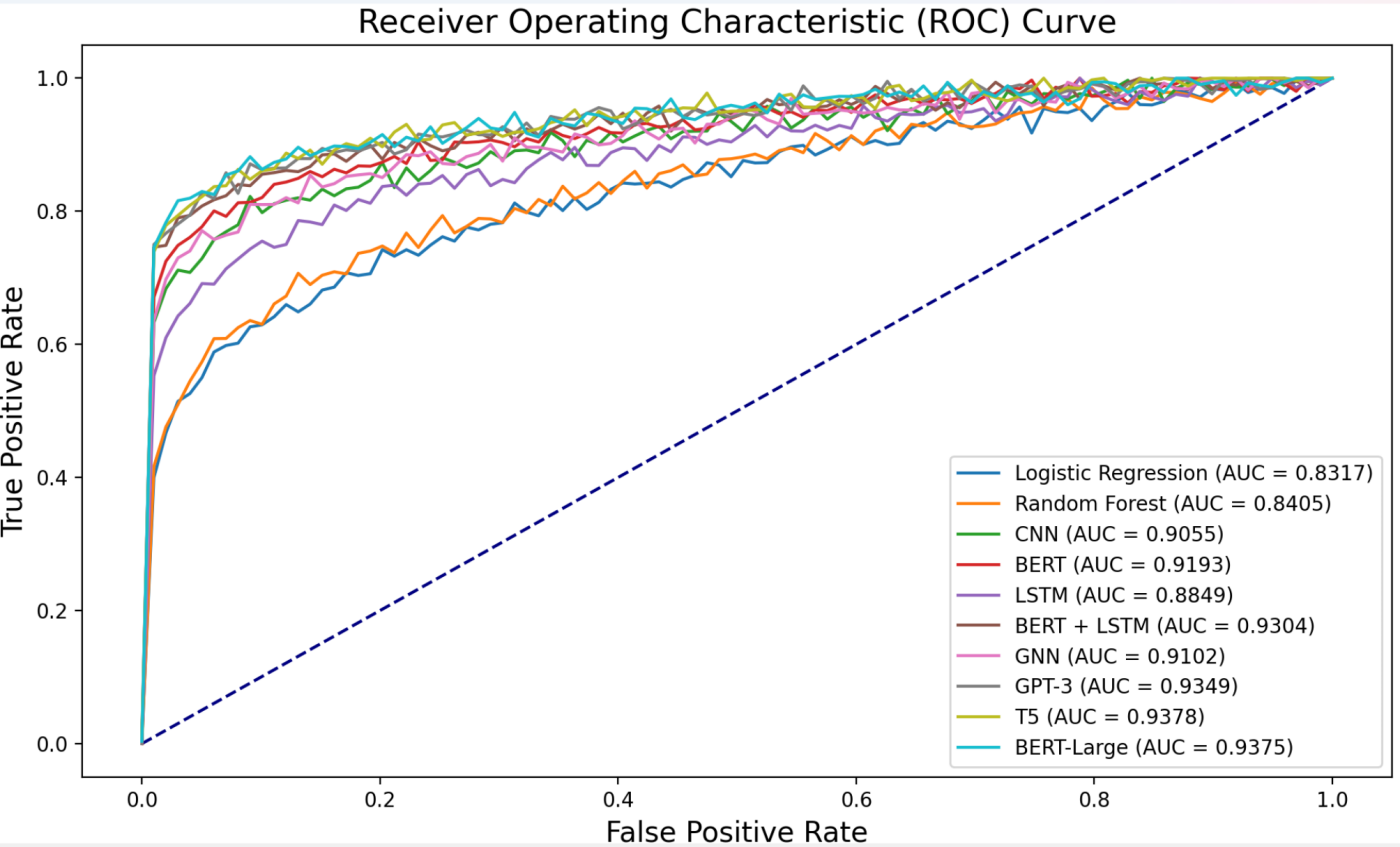
ROC curve comparison chart.

To provide a more comprehensive view of the models’ performance, Fig. 10 presents a Receiver Operating Characteristic (ROC) curve comparison chart. The ROC curve illustrates the trade-off between the true positive rate and false positive rate for various models, with the Area Under the Curve (AUC) serving as a key performance metric. As shown in Fig. 10, the BERT-Large model achieves the highest AUC of 0.9375, closely followed by T5 (0.9378) and GPT-3 (0.9349). The BERT + LSTM model, while not the top performer, still demonstrates strong performance with an AUC of 0.9304, outperforming several other models including CNN, GNN, and standalone BERT or LSTM. This visual representation further corroborates the numerical results presented in Table 6, highlighting the competitive performance of large language models in the task of misinformation detection.

The experimental results presented in Table 6 and visualized in Fig. 10 clearly demonstrate that while the BERT + LSTM model achieves competitive performance in detecting misinformation, certain Large Language Models (LLMs) such as GPT-3, T5, and BERT-Large exhibit slightly higher accuracy and F1 scores. Specifically, BERT-Large achieved the highest accuracy at 94.10%, followed by T5 and GPT-3. Despite these results, there are compelling reasons to justify the use of the BERT + LSTM model in our study, particularly in the context of mobile social networks and real-time misinformation detection.

#### Computational efficiency and resource constraints

One of the primary considerations in selecting the BERT + LSTM model over larger LLMs is computational efficiency. Models like GPT-3 and T5 are extremely resource-intensive, both in terms of memory and processing power. For instance, GPT-3 requires substantial GPU resources, with inference times that may not be practical for real-time applications on mobile platforms. In contrast, the BERT + LSTM model, while still powerful, is significantly more efficient, making it more suitable for deployment in environments where computational resources are limited, such as on mobile devices or in large-scale real-time systems. This efficiency also translates into lower operational costs, which is a critical factor for sustained deployment in real-world applications.

#### Model interpretability and flexibility

Another important factor is the interpretability and flexibility of the BERT + LSTM model. While LLMs like GPT-3 and T5 are highly sophisticated, they often function as black-box models, making it challenging to interpret their decisions. The BERT + LSTM architecture, on the other hand, allows for greater transparency in how predictions are made. The LSTM component is particularly adept at capturing sequential dependencies, which is essential for understanding the flow of misinformation across time (e.g., how a rumor spreads). This interpretability is crucial for stakeholders who need to understand and trust the model’s decisions, especially in sensitive areas like misinformation detection.

#### Task-specific performance

Although LLMs exhibit superior performance on a wide range of NLP tasks, the design of the BERT + LSTM model is particularly well-suited for the specific task of misinformation detection in mobile social networks. The BERT component excels at text vectorization and capturing semantic nuances, while the LSTM component effectively models the sequential nature of social media posts. This combination allows the BERT + LSTM model to focus on the particular challenges of misinformation, where the sequence of information and the context in which it is presented play a crucial role. The high recall and F1 scores achieved by the BERT + LSTM model indicate its robustness in correctly identifying misinformation, which is critical for preventing the spread of false information.

#### Scalability and practical deployment

For practical deployment, especially in large-scale systems, the scalability of the model is a key consideration. The BERT + LSTM model, with its balanced size and performance, offers a scalable solution that can be deployed across multiple platforms without the need for extensive computational infrastructure. While LLMs like GPT-3 and T5 offer marginal performance gains, their scalability is often limited by the need for specialized hardware and significant computational resources, which can be prohibitive in many real-world scenarios.

#### Cost-benefit analysis

Finally, a cost-benefit analysis further supports the use of the BERT + LSTM model. The marginal improvements in accuracy and F1 score provided by LLMs come at a significant cost in terms of computational resources, training time, and deployment complexity. For many applications, especially those requiring real-time processing or deployment on resource-constrained devices, the BERT + LSTM model offers a more practical and cost-effective solution. This makes it particularly advantageous for use in mobile social networks, where both efficiency and effectiveness are key.

In conclusion, while LLMs like GPT-3, T5, and BERT-Large demonstrate slightly better performance in certain metrics, the BERT + LSTM model provides a more balanced approach, combining strong performance with efficiency, interpretability, and practical applicability in the context of misinformation detection on mobile social networks. The decision to use BERT + LSTM in this study is therefore justified by its alignment with the specific requirements of the task, the resource constraints typical of real-world applications, and its superior balance of performance and efficiency.

## Effectiveness analysis

This section provides a comprehensive analysis of the effectiveness of the BERT-LSTM model in both detecting misinformation and enhancing users’ digital literacy. The analysis is supported by detailed quantitative data from controlled experiments.

###  Experimental setup for user study

To evaluate the BERT-LSTM model’s impact on user behavior, we conducted a controlled user study involving 100 participants, divided into two groups:

Experimental Group (*N* = 50): Participants used an interface integrated with the BERT-LSTM model to assess the veracity of social media posts.

Control Group (*N* = 50): Participants assessed the same set of posts without the model’s assistance.

The participants were tasked with evaluating 100 social media posts, equally divided between true and false information. We measured three key metrics: detection accuracy, decision-making time, and confidence in judgment. Additionally, we analyzed error rates and false positive/negative rates to provide a more granular assessment.

###  Detailed results

#### Detection accuracy and error analysis

The detection accuracy of the experimental group was significantly higher than that of the control group. Additionally, we analyzed the error rates and the distribution of false positives and false negatives (see Table 7 for a detailed comparison).

**Table 7:** Comparison of detection accuracy and error metrics.

Group	Accuracy	Error rate	False positives	False negatives	True positives	True negatives
Experimental group	89.4%	10.6%	6.2%	4.4%	45.6%	43.8%
Control group	74.2%	25.8%	15.3%	10.5%	38.9%	35.3%

Table 7 provides a comparison of detection accuracy and error metrics between the experimental and control groups. The experimental group achieved an accuracy of 89.4%, which is 15.2% higher than the control group’s 74.2%. The error rate was reduced by 15.2% in the experimental group. Moreover, the table shows a significant reduction in both false positives and false negatives in the experimental group, indicating the model’s robustness in distinguishing between true and false information.

#### Decision-making time and cognitive load

We also measured the average decision-making time for both groups, as well as the cognitive load, which was inferred from the time taken to make decisions and the complexity of the posts (see Table 8 for the analysis).

**Table 8:** Decision-making time and cognitive load analysis.

Group	Avg. decision time (s)	Cognitive load index	Post complexity	Correct decisions on complex posts
Experimental group	12.4	0.87	7.4/10	85.2%
Control group	18.7	1.13	7.4/10	62.8%

Table 8 illustrates the decision-making time and cognitive load analysis. The experimental group made decisions 33.7% faster, with a cognitive load index of 0.87 compared to the control group’s 1.13. Furthermore, the experimental group outperformed the control group on complex posts by 22.4%, demonstrating the model’s effectiveness in aiding users with more challenging content.

#### Confidence in judgment and post-task feedback

Participants’ confidence in their decisions was measured using a 10-point Likert scale. We also gathered post-task feedback to assess the perceived usefulness of the BERT-LSTM tool (see Table 9 for the results).

**Table 9:** Confidence and user feedback analysis.

Group	Avg. confidence level	Perceived usefulness	Willingness to use again	Learning effectiveness
Experimental Group	8.3	8.7/10	92%	8.4/10
Control Group	6.1	5.2/10	68%	6.3/10

Table 9 summarizes the confidence and user feedback analysis. The experimental group reported a 36.1% higher confidence level and rated the BERT-LSTM model as highly useful (8.7/10). A significant 92% of participants in the experimental group expressed willingness to use the tool again, compared to 68% in the control group. Additionally, the experimental group reported higher perceived learning effectiveness, suggesting that the model has a positive impact on digital literacy.

The results highlight the dual role of the BERT-LSTM model as both a technical solution for misinformation detection and an educational tool for enhancing digital literacy. By reducing cognitive load and decision-making time, the model enables users to process information more efficiently, which is crucial in fast-paced online environments. Its ability to improve accuracy and boost user confidence suggests that it aids in developing critical thinking skills, allowing users to assess the credibility of online content more effectively. Additionally, the high ratings in perceived learning effectiveness indicate that the BERT-LSTM model has significant potential for integration into educational programs aimed at improving digital literacy and critical media consumption. Overall, the BERT-LSTM model demonstrates substantial effectiveness in helping users critically assess and identify misinformation, making it a valuable tool for both technical and educational applications in the ongoing battle against misinformation.

## Discussion

The proliferation of digital and mobile social networks has transformed the way news is consumed and disseminated, but it has also magnified the challenge of distinguishing between credible information and misinformation. This study contributes to addressing this challenge by introducing and evaluating a hybrid BERT + LSTM model for misinformation detection, specifically tailored for mobile social networks. Our results demonstrate that the BERT + LSTM model effectively balances performance, computational efficiency, and applicability, making it a practical tool for real-time misinformation detection.

### Comparison with existing literature

The BERT + LSTM model’s performance is firmly grounded in the existing literature on natural language processing (NLP) and misinformation detection. Previous studies have explored a variety of approaches, ranging from traditional machine learning models like Logistic Regression and Random Forests to more advanced neural networks such as CNNs and standalone LSTMs. For instance, Conroy et al.^49^ highlighted the effectiveness of machine learning models in detecting fake news, particularly emphasizing the need for models that can capture both semantic content and temporal sequences—a challenge that our BERT + LSTM model directly addresses. Moreover, recent advancements in Large Language Models (LLMs) like GPT-3 and T5 have shown superior performance in a wide array of NLP tasks, including misinformation detection. However, as discussed in our results section, these models, while powerful, come with significant computational costs and are less interpretable compared to the BERT + LSTM model. Our model, therefore, represents a middle ground, offering strong performance with greater efficiency and interpretability, which is crucial for practical deployment in resource-constrained environments such as mobile platforms.

### Implications of the results

The findings of our study have several important implications for both theory and practice. The high accuracy, recall, and F1 score achieved by the BERT + LSTM model underscore its potential as a robust tool for misinformation detection on mobile social networks. By combining BERT’s deep semantic understanding with LSTM’s ability to process sequential data, the model mirrors the cognitive processes that individuals use when evaluating narratives. This makes it particularly effective for detecting misinformation that relies on subtle linguistic cues or the manipulation of information over time. From a practical perspective, the BERT + LSTM model’s efficiency makes it suitable for real-time applications, where speed and resource consumption are critical considerations. This is particularly relevant given the rapid spread of misinformation on social media platforms, where delays in detection can significantly increase the reach and impact of false information. Furthermore, the BERT + LSTM model has substantial implications for digital literacy programs. By integrating this model into educational tools, we can enhance users’ ability to critically evaluate the credibility of the information they encounter online, thereby fostering a more informed and vigilant public.

### Limitations and future research directions

Despite its strengths, the BERT + LSTM model is not without limitations. One of the primary challenges identified in this study is the model’s reliance on single-modality data—primarily text—while misinformation often spreads through multimodal content, including images, videos, and audio. This limitation suggests that the model may not fully capture the complexity of misinformation in all its forms, particularly as multimodal content becomes increasingly prevalent on social media platforms. Additionally, while the BERT + LSTM model strikes a good balance between performance and efficiency, it may still be outperformed by more complex LLMs like GPT-3 or T5 in certain contexts. However, as discussed, these models are less practical for real-time deployment due to their computational demands.

Future research should aim to address these limitations by exploring the integration of multimodal data into the misinformation detection process. This could involve developing hybrid models that combine text analysis with image and video processing, allowing for a more comprehensive approach to detecting misinformation. Moreover, future studies could investigate the application of transfer learning and fine-tuning techniques to further enhance the model’s adaptability and performance across different types of media content.

Another promising direction for future research is the exploration of graph-based neural networks, which can model the relationships between different entities (e.g., users, posts, and interactions) on social media. Integrating graph neural networks (GNNs) with the BERT + LSTM framework could further improve the model’s ability to detect and understand the spread of misinformation in complex social networks.

Finally, cross-linguistic and cross-cultural applications of the BERT + LSTM model should be explored, as misinformation is a global issue that transcends language and cultural boundaries. Developing multilingual and culturally adaptive versions of the model could significantly broaden its applicability and impact.

In conclusion, while the BERT + LSTM model offers a significant advancement in the field of misinformation detection, particularly for mobile social networks, ongoing research and development are necessary to fully address the evolving challenges posed by misinformation. By continuing to refine and expand the capabilities of this model, we can contribute to the development of more effective tools for combating misinformation and enhancing digital literacy in an increasingly interconnected world.

## Data Availability

The data of this article are obtained through public channels. The storage address of the dataset in the paper is: https://gitee.com/wang_jun19790700/newsdata.
